# Give Blood 4 Good: Improving Blood Donation Statistics in Young People

**DOI:** 10.7759/cureus.92133

**Published:** 2025-09-12

**Authors:** Alexandra C Davies, Gemma M Smith

**Affiliations:** 1 Medicine, University Hospital of North Durham, Durham, GBR; 2 Stroke Medicine, University Hospital of North Durham, Durham, GBR

**Keywords:** charity, leukaemia, public health education, public health policy, voluntary blood donation

## Abstract

Thousands of blood donations are needed every day in the United Kingdom (UK). Give Blood 4 Good is on a mission to improve blood donation statistics through education and awareness of blood donation, particularly in young people. This editorial discusses the daily blood donation requirements in the UK and some of the barriers holding individuals back from donating blood. The editorial discusses a patient and their experience with blood donation, their illness, and Give Blood 4 Good, a charity that aims to increase blood donation in the UK. Increasing awareness of blood donation and its life-extending ability is vital in removing the fear that so many people associate with blood donation and encouraging a lifetime habit of regular donation from a young age.

## Editorial

What is the blood donation situation in the United Kingdom (UK)? On average, 4,300 blood donations are needed every day in the UK [1]. In 2022, a major UK survey demonstrated that only 7% of adults are regular donors, and 58% stated that they had never donated blood before [2]. The number of individuals aged 18-24 who had never donated blood was extremely high at 70%; only 11% of this age group were regular donors [2]. NHS Blood and Transplant data shows that although the total number of active blood donors increased in 2022/2023 to just under 800,000 [1], there is still a long way to go. Give Blood 4 Good is on a mission to improve these statistics by increasing the number of blood donors, particularly young adult donors, through #BloodyBrilliant university teams, Young Ambassador Programmes in schools, and social media campaigns that spread the word about blood donation.

Often, individuals are unable to donate blood due to medical reasons, such as anaemia or having received a previous blood transfusion [3]. However, there are still vast numbers of eligible donors who do not donate, particularly in younger age groups [2]. There are many reasons that people give for not donating blood: two key reasons often given are never having considered it and not getting around to donating [2]. Neither reason should ever be a barrier to donation. NHS England data demonstrated that fear of blood donation is another significant barrier; 35% of 18-24-year-olds have admitted that they have not donated due to being scared [4]. A barrier of particular relevance to the lesbian, gay, bisexual, transgender, queer, intersex, asexual, and plus (LGBTQIA+) community is the lack of awareness about the changes made in 2021, following the FAIR report [5], which now allow individuals to donate blood regardless of the gender of their sexual partner [5]. Give Blood 4 Good is working to address all of these barriers.

In October 2019, an 18-year-old woman was diagnosed with acute myeloid leukaemia (AML), a type of blood cancer, whilst studying at university. Over the next three and a half years, this woman went through many intensive treatments: bone marrow transplants, chemotherapy, and infusion of blood products. Both AML and its treatments cause the number of blood cells to decrease [6], meaning she relied on regular blood transfusions to keep her red blood cells and platelets high enough [6]. Throughout the course of her illness, she received over 100 transfusions as a part of her treatment; many times after receiving a transfusion, she described feeling brighter and more energised, something her family regularly noticed too. Without these transfusions, her treatment would not have been possible; she was utterly reliant on donated blood to survive.

Sadly, this young woman passed away in May 2023, but the hundreds of transfusions she received over three and a half years gave her the energy and ability to do what was essential to her during her treatment. This included completing her degree and graduating from university, raising awareness of blood cancer online, having adventures in the outdoors with her loved ones, and adopting a beloved rescue dog. In honour of what would have been her 23rd birthday, Give Blood 4 Good organised a blood drive with this young woman’s friends and family to encourage people to donate blood, so that others can benefit from the same treatments she received. Over the course of the drive, 230 people pledged to donate blood in her memory, and 116 were first-time donors. These pledges to donate blood saved or improved the lives of up to 690 individuals [7].

Give Blood 4 Good is a charity founded in 2019 by Hanna Smith and Martha Greenbank. Its mission is to encourage as many young adults as possible across the UK to donate blood, to ensure that everyone who needs a blood transfusion can receive one, whilst also leaving behind a bright legacy in memory of Hanna’s brother, Patrick. Patrick, who lost his life in an accident at the age of 21, was highly passionate about donating blood, so much so that he donated on the first day he was eligible, on his 17th birthday. Following a blood drive held in his honour, in which over 150 people donated blood, we realised that 60% of those who took part had never given blood before and wouldn’t have considered it without the blood drive [8]. We discovered that the majority of those aged 18-24 in the UK had never donated blood [8]. This highlighted to us that more must be done to improve education surrounding blood donation in young adults. Give Blood 4 Good was formed to do exactly that.

Our #BloodyBrilliant University Squads (BBUS) have volunteer teams at major Scottish universities. We work within those universities and also work in partnership with other universities across the UK, such as the University of Manchester and the University of Cambridge. Our volunteers improve education and increase participation in blood donation amongst young adults through organising blood drives, fundraising events, publishing articles, and much more. The #BloodyBrilliant impact of Give Blood 4 Good’s university teams is clear to see: in the 2023/2024 academic year, they encouraged almost 1000 young people to pledge to donate blood, of which 64% were first-time donors. Many of these donors would never have considered donating blood without the interventions of Give Blood 4 Good. The actions of our charity’s volunteers increase awareness and empower young people to donate and, hopefully, continue to make regular donations for life.

Alongside our BBUS, Give Blood 4 Good also works in schools. We educate young people on the importance of blood donation through our e-learning platform, where students learn about when people need transfusions, which blood types can be given, and what giving blood actually involves. In this way, Give Blood 4 Good is breaking down misconceptions around blood donation to remove the fear that many young people associate with it. In 2024/2025, Give Blood 4 Good will be increasing the number of schools and universities that we work with to further circulate the word of blood donation.

In addition to the Young Ambassador Programme and BBUS, Give Blood 4 Good’s ever-growing social media presence is fostering discussions around blood donation through articles and stories about individuals who have received blood transfusions. Our donor stories discuss transfusions during childbirth, leukaemia, sickle cell disease, and more. Sharing these stories demonstrates to people the importance of donating blood; you never know when you or a loved one may need a blood donation.

This editorial is critical, and we hope that by discussing and reflecting on this young person's journey through her illness and the vital role that blood donation played in enabling a meaningful quality of life in her final year, we can raise awareness about the importance of blood donation. Increasing awareness and removing the stigma surrounding blood donation can help improve the availability of blood products and assist individuals, such as the young person in this case study. Increased awareness is particularly vital in the current climate, where blood services are struggling to meet the current demands.

Alongside increasing the number of schools and universities that Give Blood 4 Good works with, the next steps for the charity include holding more events and fundraisers highlighting the importance of blood donation, sharing more donor stories, and extending the organisation of blood drives into the corporate sphere. Give Blood 4 Good is a small charity, and some limitations that we face include volunteer time availability and geographical distance from other team members for collaboration. Although Give Blood 4 Good works closely with NHS Blood Donation services, there are also complex data-sharing protections that can make measuring specific impact factors complex. Give Blood 4 Good is striving towards the aim of improving blood donor statistics in young people across the UK. Through education of young people, organisation of blood drives, and efforts to spread the word of blood donation, we will continue on our mission.
